# Abiraterone-Associated Mineralocorticoid Excess: A Case Report

**DOI:** 10.7759/cureus.51757

**Published:** 2024-01-06

**Authors:** Saeed K Shaffi, Raja Ravender, Chandra Kumar Mallick Kodavanti, Brent Wagner, Manoocher Soleimani

**Affiliations:** 1 Nephrology, Raymond G. Murphy Veterans Affairs Medical Center, Albuquerque, USA; 2 Nephrology, University of New Mexico School of Medicine, Albuquerque, USA; 3 Critical Care Medicine, Southeast Health Medical Center, Dothan, USA

**Keywords:** abiraterone induced hypokalemia, abiraterone induced metabolic alkalosis, abiraterone induced hypertension, syndrome of apparent mineralocorticoid excess, abiraterone acetate

## Abstract

Abiraterone acetate causes an adrenocorticotropic hormone (ACTH)-mediated mineralocorticoid excess. We present a 77-year-old man with prostate adenocarcinoma who developed signs and symptoms of mineralocorticoid excess while on abiraterone and discuss its pathophysiology and treatment options. The patient developed hypokalemia, metabolic alkalosis, and hypertension, indicative of increased mineralocorticoid activity, confirmed by elevated ACTH, corticosterone, and deoxycorticosterone levels. Abiraterone inhibits cytochrome P450c17 (CYP17A1), thus inhibiting testosterone and cortisol synthesis. Diminished cortisol synthesis, in turn, leads to excessive mineralocorticoid precursor production mediated by ACTH, leading to enhanced sodium absorption and potassium excretion. Abiraterone is often prescribed with low-dose prednisone to suppress ACTH; however, this strategy may not provide physiological glucocorticoid levels, resulting in ACTH-mediated mineralocorticoid excess in some patients. High-dose steroids or mineralocorticoid antagonists may activate mutant androgen receptors in prostate cancer tissue; therefore, amiloride is suggested for managing residual mineralocorticoid activity. This case highlights the importance of being vigilant for the signs and symptoms of mineralocorticoid excess in patients on abiraterone.

## Introduction

Abiraterone acetate, an inhibitor of testosterone and its precursors', has been approved for treating prostate cancers (castration-resistant or sensitive) when used in conjunction with prednisone [1,2]. Abiraterone causes mineralocorticoid excess, mediated by adrenocorticotropic hormone (ACTH), by inhibiting cortisol synthesis in the adrenal zona fasciculata [3]. This diversion of the pathway leads to the production of corticosterone and deoxycorticosterone, both exhibiting mineralocorticoid activity. Reduced serum cortisol levels subsequently trigger ACTH release, driving the synthesis of compounds with mineralocorticoid traits. These compounds foster fluid retention, kaluresis, hypokalemia, chloride-resistant metabolic alkalosis, and eventual hypertension. Prednisone supplementation addresses these concerns by reinstating a physiological serum glucocorticoid concentration, curbing ACTH production, and thus interrupting the vicious cycle of low cortisol, excessive ACTH, fluid retention, and hypertension [3].

Nevertheless, a subset of abiraterone-treated patients still encounters mineralocorticoid excess despite concurrent prednisone use [1,2]. In this context, we present a patientwho developed abiraterone-associated mineralocorticoid excess and delve into the underlying pathophysiology, the diagnostic strategies employed, and the available treatment options.

## Case presentation

A 77-year-old man with an elevated plasma prostate-specific antigen (PSA) level was diagnosed with prostate adenocarcinoma. A bone scan showed diffuse bony metastasis with an incidental right lung nodule, which, on lung biopsy, was an invasive non-small cell adenocarcinoma. For prostate cancer, urology initiated anti-androgen therapy (bicalutamide 50 mg orally once daily and leuprolide 45 mg subcutaneously every 24 weeks). Approximately two and a half months later, abiraterone one gram daily was also initiated. Prednisone 5 mg daily was added to the regimen about three months later. Due to mediastinal involvement, he was not a surgical candidate; therefore, the lung adenocarcinoma was treated with a dual chemotherapy regimen of carboplatin and pemetrexed with concurrent radiation. After the first chemotherapy cycle, he reported fatigue and poor appetite. His performance status had decreased; therefore, the second chemotherapy session was withheld, but he continued with the radiation therapy. 

**Figure 1:**
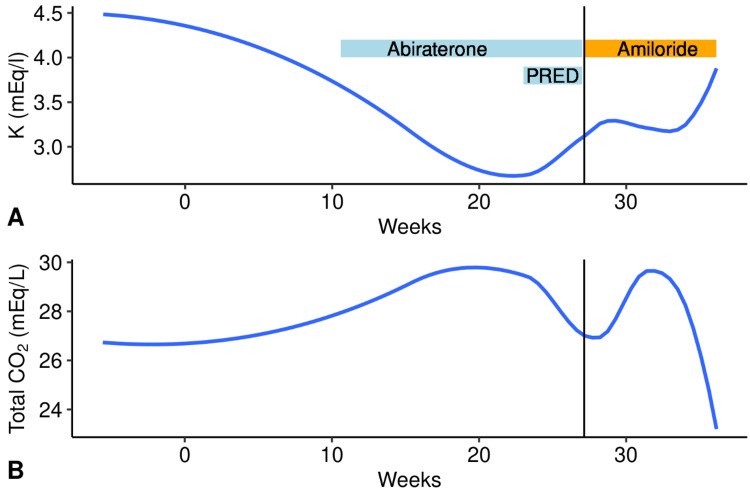
Graphs showing (A) plasma potassium and (B) total carbon dioxide (both loess smoothed) over weeks since diagnosis The patient was diagnosed with prostate cancer at week 0. The effects of abiraterone with and without prednisone (PRED) administration on plasma K and total carbon dioxide (CO_2_) are also shown (light-blue rectangles). The vertical line represents the hospitalization time point. The initiation of amiloride (orange rectangle) resulted in an improvement in plasma K and total CO_2_.

Figure 1 shows his plasma potassium levels during this time. Within two months of chemotherapy initiation, his plasma potassium level had declined to 2.4 mEq/l, which was treated with oral potassium replacement. Despite replacement, his plasma potassium remained low; consequently, the potassium chloride (KCl) dose was increased. Additionally, he had numerous KCl infusions without significant plasma potassium improvement. Six months after chemotherapy initiation, his plasma potassium was 1.9 mEq/l, for which he was hospitalized. 

On nephrology assessment, the patient had malaise, nausea, and poor appetite. He also had diarrhea with up to six watery bowel movements daily. His legs were chronically swollen. He was living alone and had difficulty performing his daily activities. Pertinent findings on examination were blood pressure (BP) of 180/84 mmHg, a pulse of 75 beats per minute, oxygen saturation (SPO_2_) of 93% on room air, and 2+ lower extremities pitting edema. His lungs, cardiovascular, and abdominal examinations were unremarkable. Arterial blood pH was 7.46 with a plasma total carbon dioxide (CO_2_) of 32 mEq/l. The rest of the electrolytes are shown in Table 1. A spot urine potassium-creatinine (K/Cr) ratio was 206 mEq/g, indicating urinary potassium wasting (Table 2). After K replacement, his plasma renin activity and plasma aldosterone concentration were assessed, which were not consistent with primary hyperaldosteronism or renal artery stenosis (Table 1). A random plasma cortisol level assessed at 8 AM was not elevated (10 micrograms/dl). The possibility of abiraterone-associated ACTH-mediated syndrome of mineralocorticoid excess was entertained. After consultation with oncology, abiraterone was discontinued, and we started the patient on amiloride at 5 mg orally daily, with improvement in plasma K and a decline in plasma total CO_2_ (Figure 1). An elevated plasma ACTH, corticosterone, and deoxycorticosterone (Table 1) confirmed an ACTH-mediated state of mineralocorticoid excess, which most likely was due to abiraterone but could also be due to ectopic ACTH secretion by the lung adenocarcinoma. 

**Table 1:** Selected laboratory data around the time of admission * showing elevated levels ACTH - adrenocorticotropic hormone; CO_2_ - carbon dioxide

Variable	Value
Cortisol (ug/dl)	10
ACTH (pg/ml; AM reference range 6-50)	220*
Plasma aldosterone concentration (ng/dl)	<1
Plasma renin activity (ng/ml/hr)	0.13
Corticosterone (ng/dl; AM reference range: 59-1293)	>10,000*
Deoxycorticosterone (ng/dl; reference range ≤16)	100*
Plasma total CO_2_ (mEq/l)	32*
Arterial pH	7.46*

**Table 2:** Random urine potassium and creatinine (K/Cr) ratios (mEq/g) and plasma potassium (K) during admission

Days after abiraterone discontinuation and on amiloride therapy	K/Cr (mEq/g)	Plasma K (mEq/l)
0	206	2.5
4	59	3.9

Unfortunately, his clinical status worsened, and he decided to go on hospice care about eight and a half months after the prostate cancer diagnosis.

## Discussion

**Figure 2:**
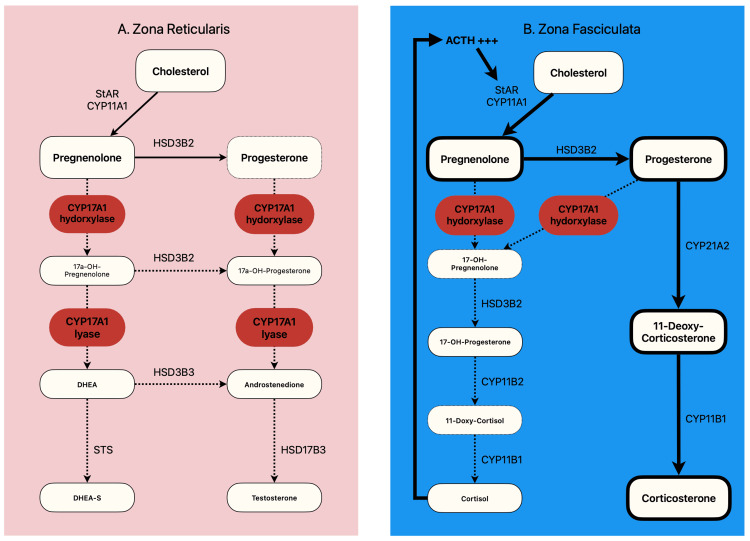
Steroid synthesis pathways in the adrenal zona reticularis (A) and fasciculata (B) (A) Abiraterone inhibits CYP17A1 enzyme complex (hydroxylase and lyase) in the zona reticularis with almost complete cessation of testosterone and its precursors production. Red boxes and Interrupted arrows signify enzyme inhibition and pathway blockade, respectively. (B) In the zona fasciculata, inhibition of CY17A1 hydroxylase results in decreased cortisol and increased 11-deoxycorticosterone and corticosterone production via the back door pathway. A low cortisol level triggers ACTH release, causing the propagation of this vicious loop. These processes are represented by dark arrows and highlighted boxes. StAR - steriodogenic actor regulatory protein; CYP11A1 - cytochrome P450 family 11 subfamily A member 1; CYP17A1 - cytochrome P450 family 17 subfamily A member 1; CYP21A2 - cytochrome P450 family 21 subfamily A member 2; CYP17A lyase - cytochrome P450 17alpha-lyase; CYP11B1 - cytochrome P450 family 11 subfamily B member 1; HSD3B2 - hydroxy- delta-5-steriod dehydrogenase 3 beta- and steroid delta-isomerase 2; HSD17B3 - hydroxysteroid 17-beta dehydrogenase 3; 17-OH progesterone - 17α-hydroxyprogesterone; DHEA - dehydroepiandrosterone; DHEA-S - dehydroepiandrosterone sulfate; STS - steroid sulfatase; ACTH - adrenocorticotropic hormone

Abiraterone acetate is a drug that selectively and irreversibly inhibits cytochrome P450c17 (CYP17A1) with the resultant suppression of 17 alpha-hydroxylase and C17,20-lyase [3]. These enzymes are expressed in the testicular and adrenal glands and prostate cancer tissues. In the Zona Reticularis, inhibition of CYP17A1 (hydroxylase and lyase) leads to decreased synthesis of testosterone and its precursors dehydroepiandrosterone (DHEA), dehydroepiandrosterone sulfate (DHEA-S), and androstenedione (Figure 2A). These anti-androgenic effects are primarily responsible for the mortality benefit in patients with prostate cancer.

**Figure 3:**
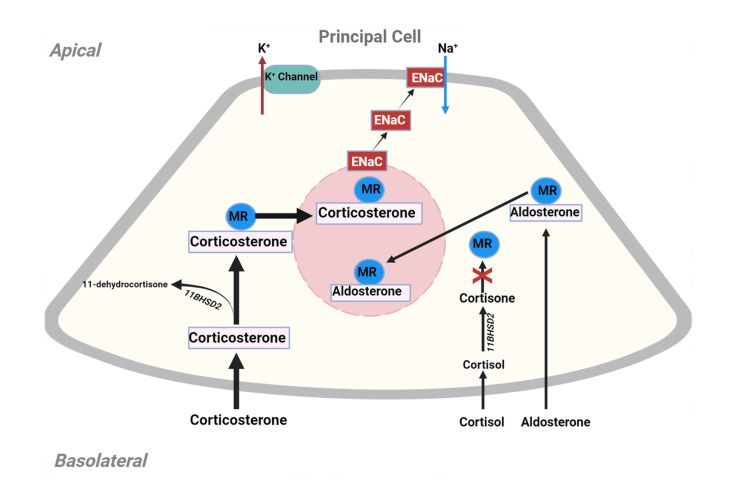
Mineralocorticoid effects in the principal cell Mineralocorticoids (aldosterone, cortisol, or corticosterone) bind with the cytoplasmic mineralocorticoid receptor (MR; represented as blue circles) with equal affinity. However, 11 beta-hydroxysteroid dehydrogenase 2 (11BHSD2) converts cortisol and corticosterone to cortisone and 11-dehydrocortisone, respectively - compounds that lack the ability to bind with the MR receptor (red cross). Abiraterone causes ACTH-triggered large amounts of corticosterone production, which overwhelms 11BHSD2, resulting in its uninhibited binding with the MR. Corticosterone-bound MR is internalized in the nucleus, leading to transcription of the epithelial Na channels (ENaCs; red rectangles) and their insertion on the luminal side of the principal cell. ENaCs enable Na absorption, which, in turn, generates an intra-luminal negative charge and facilitates K efflux via the renal outer medullary K (ROMK) channels (green box). ACTH - adrenocorticotropic hormone

The zona fasciculata of the adrenal cortex - the site of cortisol synthesis - expresses CYP17A1 hydroxylase but lacks CYP17A1-lyase. Abiraterone-mediated inhibition of CYP17A1 hydroxylase prevents the conversation of pregnanolone to cortisol; instead, the pathway is shunted towards the formation of 11-deoxycorticosterone and corticosterone, which have mineralocorticoid activity (Figure 2B). Cortisol deficiency drives excessive ACTH synthesis by a feedback mechanism that amplifies mineralocorticoid production by the zona fasciculata (Figure 2B). Abiraterone does not significantly affect the zona glomerulosa of the adrenal cortex, which lacks the CYP17A1 enzyme complex. The ensuing elevated 11-deoxycorticosterone and corticosterone enhance salt absorption and potassium excretion in the kidney collecting duct (Figure 3), therefore increasing the systemic blood pressure and causing hypokalemia, which suppresses aldosterone by a feedback mechanism.

Our patient presented with diarrhea and nausea, which were potentially caused by chemotherapy. On presentation, he was hypokalemic, which could have been worsened by diarrhea. However, he had metabolic alkalosis instead of a non-anion gap metabolic acidosis, which is typically seen in diarrhea. Furthermore, the urine K/Cr ratio was elevated, pointing toward renal potassium wasting (Table 2). He was hypertensive with signs of volume overload, along with an elevated plasma total CO_2_ (Table 1). These findings raised the possibility of mineralocorticoid excess. After normalizing his potassium with intra-venous potassium supplementation, plasma renin and aldosterone levels were assessed, which were not elevated, therefore excluding the possibility of primary hyperaldosteronism or renal artery stenosis (Table 1). His random plasma cortisol level was not abnormal either; however, a low or a high dose dexamethasone suppression test was not performed. Mineralocorticoid excess, as the cause of these abnormalities, was confirmed by elevated plasma levels of ACTH, corticosterone, and deoxycorticosterone. 

In this patient, the source of ACTH-driven mineralocorticoid excess could have been due to pituitary or ectopic ACTH production vs. abiraterone. Pituitary adenomas can cause Cushing's disease, which is associated with elevated ACTH and cortisol levels. A supra-physiological dose of dexamethasone administration may suppress the pituitary-derived ACTH, which is the rationale for its use to differentiate pituitary vs. ectopic ACTH secretion; however, this phenomenon is not seen in all the pituitary adenomas [4]. ACTH can also be secreted by neuro-endocrine malignancies of various organs; nonetheless, the small-cell carcinomas of the lung are by far the most common cause of malignancy-associated ectopic ACTH secretion. They are unlikely to show cortisol suppression in response to a high-dose dexamethasone administration test. This patient had lung cancer, which could have been the source of the elevated ACTH. However, various lines of evidence suggest that abiraterone was the cause of aberrant ACTH secretion. Firstly, there was a temporal association between abiraterone administration and the development of hypokalemia (Figure 1) and hypertension. Secondly, prednisone administration improved plasma potassium modestly, even in the absence of amiloride administration (Figure 1). Lastly, serum potassium, hypertension, and volume overload improved significantly following abiraterone's discontinuation and amiloride's addition.

Abiraterone improves overall survival in patients with castration-resistant metastatic prostate cancer [1,2]. In the phase I clinical trial, abiraterone administration resulted in a significant decrease in testosterone and an increase in corticosterone and deoxycorticosterone [5]. Coadministration of dexamethasone 0.5 mg/day, a potent glucocorticoid, to abiraterone acetate regimen suppressed corticosterone and ACTH levels to less than two and three-fold, respectively. Considering these results, in the phase II trial of abiraterone in metastatic castration-resistant prostate cancer, abiraterone 1000 mg daily was co-administered with 10 mg of prednisone in two divided doses in the treatment group [6]. The adverse events of fluid retention, hypertension, and hypokalemia were encountered less often in the coadministration group; therefore, in 2011, FDA approved abiraterone acetate with prednisone 5 mg twice a day for metastatic castration-resistant prostate cancer[1]. Based on a subsequent study that showed effective control of symptoms of mineralocorticoid excess when abiraterone was co-administered with 5 mg of prednisone, FDA-approved abiraterone with a lower dose of prednisone for patients with high-risk castration-sensitive prostate cancer [2]. A review of that study reveals that approximately one in three patients developed the adverse event of hypertension (37%), and one in five developed hypokalemia (20%) on abiraterone with 5 mg of prednisone. However, none of the patients developed severe adverse events or required prednisone dose escalation.

In patients on abiraterone, prednisolone or its derivates can normalize ACTH levels. However, an adequate plasma glucocorticoid concentration is necessary to suppress ACTH secretion. In a study on 15 castration-resistant prostate cancer patients on abiraterone with 10 mg of prednisolone, a median prednisolone concentration of 152 nmol/L was achieved, which is equivalent to 608 nmol/L of cortisol [7]. Cortisol follows a circadian rhythm with a peak concentration around 8:30 AM of 399 nmol/L with a nadir of <50 nmol/l at 2 AM [8]. Therefore, 10 mg of prednisolone can achieve physiological glucocorticoid levels in most patients on abiraterone and thus suppresses ACTH. However, some patients in that study achieved a prednisolone level of <75 nomol/l, which may be due to inducers of or intra-subject variability in cytochrome P450 3A4 (CYP3A4) activity resulting in increased prednisolone clearance [9]. Prednisone, a prodrug of prednisolone, is activated by the enzyme 11B-hydroxysteroid dehydrogenase. Patients with liver failure may have decreased or slow conversion of prednisone to its active form, resulting in low plasma glucocorticoid levels. For these reasons, up to 55% of patients on abiraterone with prednisone/prednisolone develop side effects related to mineralocorticoid excess [1]. 

Various mineralocorticoid antagonists have been used to suppress the residual mineralocorticoid activity in patients on abiraterone with low-dose steroids. However, this approach has been associated with the concern that some of the mineralocorticoid antagonists and glucocorticoids may mutate androgen receptors in the prostate cancer tissue, which could lead to treatment-resistant progressive cancer [7]. Spironolactone, eplerenone, and glucocorticoids have been shown to bind and activate both wild-type and mutant androgen receptors; therefore, their use to palliate mineralocorticoid excess-mediated symptoms is discouraged [5,7]. Given the above limitations, and based on the physiological impact of compounds with mineralocorticoid activity on the epithelial sodium channel (ENaC) at the aldosterone-sensitive distal tubules, the inhibition of ENaC would provide significant protection against sodium retention and kaluresis (Figure 3). Amiloride - a specific inhibitor of ENac - has been successfully utilized to manage the symptoms of mineralocorticoid excess in patients on abiraterone with prednisone without affecting its inhibition of prostate cancer cells [10] and thus allows continued use of this effective therapy. 

## Conclusions

This case presentation highlights ACTH-driven excessive mineralocorticoid production, which was likely due to abiraterone. The use of prednisone or prednisolone suppresses ACTH; however, a physiological serum glucocorticoid level is necessary to achieve this. High doses of glucocorticoids and mineralocorticoid antagonists may activate the mutant androgen receptors and should be avoided. Amiloride may be an effective alternative in managing the residual mineralocorticoid activity in patients who are on abiraterone. 
